# A call for minimum standards in methodological reporting for studies using animal-borne accelerometers

**DOI:** 10.1186/s40317-026-00506-9

**Published:** 2026-10-16

**Authors:** Daire Carroll, Jessica Harvey-Carroll, Jose Luis Crespo-Picazo, Daniel García-Párraga, David March

**Affiliations:** 1 Department of Biological and Environmental Sciences, University of Gothenburg, Gothenburg, Sweden; 2 Gothenburg Global Biodiversity Centre, Gothenburg, Sweden; 3 Conservation Department, Fundación Oceanografic de la Comunitat Valenciana, Valencia, Spain; 4 Cavanilles Institute of Biodiversity and Evolutionary Biology, University of Valencia, Valencia, Spain; 5 Centre for Ecology and Conservation, University of Exeter, Exeter, UK

**Keywords:** Biologging, Accelerometers, Welfare, Behaviour, Sea turtle, Replicability, Transparency, Attachment methods, Activity tracking

## Abstract

Animal-borne tri-axial accelerometers are increasingly used to investigate animal behaviour and energetics. Critical methodological details, such as device placement and dynamic range, are frequently underreported. This lack of reporting hampers study replicability and interpretation of results. It also complicates comparisons between studies. In addition, as device placement and attachment method can impact animal welfare and survival, transparent reporting should be considered vital for ethical publication. In this comment, we highlight these issues through a review of methodological reporting in studies involving attaching accelerometers to sea turtles. To address these challenges, we propose a framework for minimum standardised reporting that outlines key metadata which researchers should include when publishing studies utilizing animal-borne accelerometers. We also recommend thorough testing of devices and attachment methods prior to their use in the field. Adoption of this framework will enhance transparency, reproducibility, and comparability across studies, while promoting best practices in animal welfare.

**Supplementary Information:**

The online version contains supplementary material available at https://doi.org/10.1186/s40317-026-00506-9.

## Background

Ecological research is facing a well reported reproducibility crisis [1, 2]. Lack of methodological transparency is a contributing factor [2, 3]. This issue extends to the field of biologging, where the use of animal-borne data-logging devices, such as GPS tags and time–depth recorders, has revolutionised our ability to study free-ranging animals in their natural environments [4–8]. In part due to the field’s rapid advancement, biologging studies often suffer from inconsistencies in data collection and methodological reporting protocols [6, 8–10]. This can prevent replication as well as meaningful interpretation of results and may be an issue in subsequent meta-analysis [10, 11]. Negative effects of biologgers on animal welfare and survival have been reported across a range of taxa [7, 12–15]. To understand potential negative effects of biologgers, and limit them in future work, proper methodological reporting is essential [7, 16, 17].

In response, there have been general calls for the standardisation of reporting, with the goal of improving reproducibility, ensuring robust ecological inference, and reducing negative welfare and survival outcomes [7, 8, 10, 17, 18]. Bodey et al. [7], for example, made a general call for the standardising the reporting of biologging studies. More recently, Payne et al. [10] proposed a machine-readable checklist to streamline reporting and facilitate future meta-analysis while reducing negative impacts on animal welfare. Given the wide range of functions and study systems for which biologger devices are used, these previous calls for standardisation of reporting have been necessarily quite general. They have included parameters such as study species, number of devices deployed, number of tagged animals, mean animal mass, method of attachment, device mass, and length of deployment. Both Bodey et al. [7] and Payne et al. [10] pointed out that, beyond these general parameters, specific device types/functionalities likely have their own reporting needs.

Given the rapid pace of development in biologging, we argue that more specialised minimum reporting standards are needed for specific biologging methodologies and use cases. Here, we focus on animal-borne tri-axial accelerometers (hereafter, accelerometers). Accelerometers are an increasingly popular class of biologging device. Even within the relatively young field of biologging, the use of accelerometers remains in its infancy, with early studies published in the 1990s, followed by a sharp increase in recent years [6]. Despite this exponential growth, relatively little effort has yet been directed toward replicating accelerometer-based studies, with priority often given to deploying tags on new species rather than revisiting previously studied systems [6, 18]. Numerous study systems (different species and device models) have been developed in parallel, and only a limited number of meta-analyses have utilised accelerometer data [7, 19].

We recently attempted to collate device settings from accelerometers used in sea turtle studies and identified substantial gaps in the reporting of multiple metrics. We anticipate that such under-reporting will become an increasing problem as accelerometer use matures in biologging and the need for replication and meta-analysis grows, similar to the well reported problems in other areas of biologging [6, 10, 19]. To address this issue, we outline the specific reporting requirements of accelerometer studies beyond those of general biologging research and propose new minimum reporting standards for accelerometers.

### Accelerometers as biologging devices

Tri-axial accelerometers are biologging devices that record acceleration (the rate of change in velocity with respect to time) along three orthogonal axes (generally referred to as heave, sway, and surge) [20]. Accelerometers continuously measure static acceleration (resulting from gravity acting on the sensor) and dynamic acceleration resulting from motion [6, 21].

Accelerometers facilitate insight into previously inaccessible aspects of animal’s daily lives [4, 6, 22]. In the simplest cases, behavioural events can be inferred through threshold-based segmentation of the data, or peak detection [23–25]. Data can also be processed to estimate summary metrics (e.g., mean, maximum, and minimum acceleration along each axis) and Dynamic Body Acceleration (DBA, often used as an index of overall energy expenditure) [26, 27]. Together, these metrics can be used to infer specific behaviours, with classification often automated using machine learning [19].

**Fig. 1:**
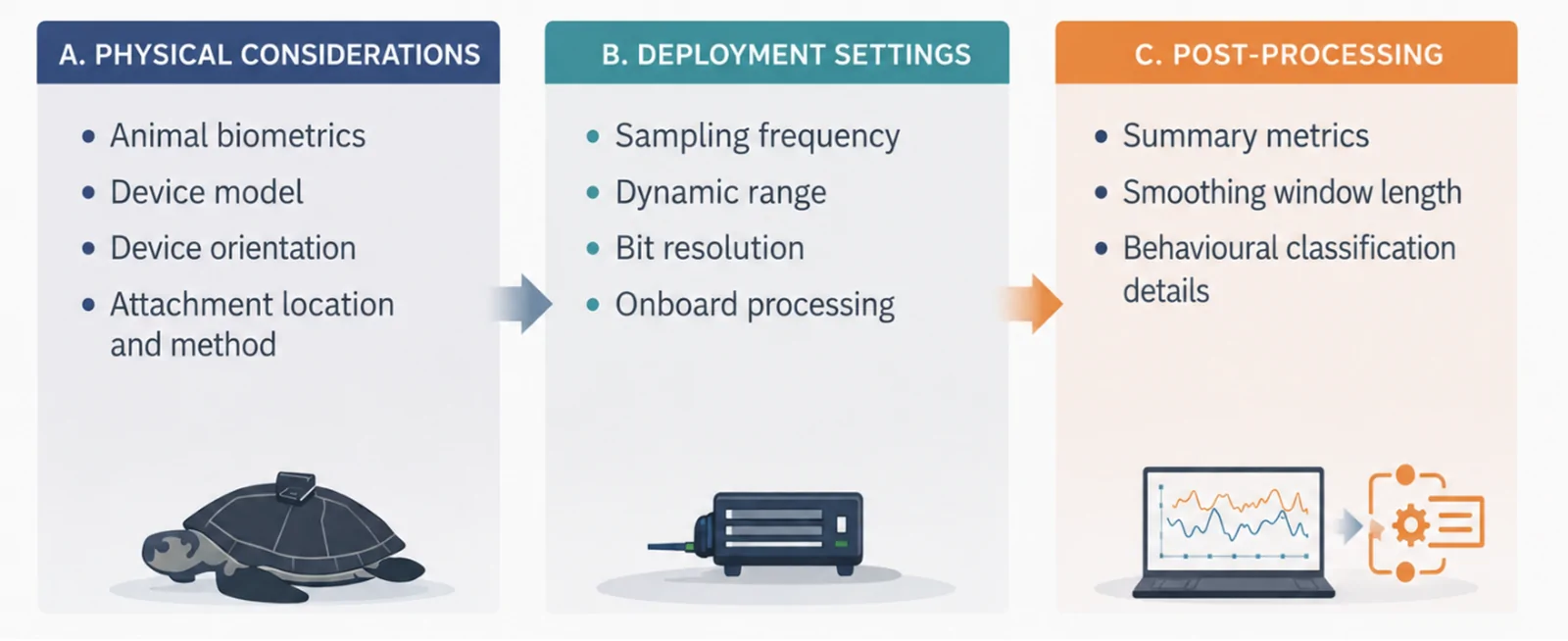
Conceptual diagram of the specific considerations for reporting in accelerometers

Accelerometers are becoming increasingly popular in research [6, 28–30]. Despite their increasing affordability and widespread use, there is a lack of standardisation in accelerometer attachment protocols and device settings within and between study species [6, 22, 28]. There is often no justification given for choice of study design and settings used [6]. The analysis of accelerometer data often involves several postprocessing steps, each of which involve deliberate choices which might not be evident to readers who have not worked in the study system [26]. A range of physical considerations, deployment settings, and data post processing can all influence the interpretation of accelerometer outputs and study replicability. As a result, accelerometer studies have specific reporting needs beyond the general considerations applicable to all biologging studies (Fig. 1) [6, 7].

### Physical considerations

Individual animal characteristics (**biometrics**), such as species, size, life-history stage, and sex are likely to influence movement kinematics (e.g., stride or wingbeat frequency) [31, 32], and thereby the magnitude and variability of acceleration recorded. These characteristics will also have a large influence on the range of behaviours an individual performs. Similarly, the captivity status of the animal will influence behaviour as captive individuals may exhibit a restricted or atypical behavioural repertoire compared to free-ranging conspecifics [22, 30].

Different **device models** may produce different raw signals even under similar conditions because of differences in sensor design and onboard processing [33]. For example, Mizrahy-Rewald et al. (2023) found that the shape of biologging devices impacted both heart rate and calculated VeDBA values for the Northern Bald Ibises (*Geronticus eremita*) [34].

Several notable studies have involved developing novel devices [35, 36]. We suggest that reporting the exact make, model, and firmware of devices is a minimum for replication of a study. In the case of custom devices, a complete technical description sufficient for a qualified individual to replicate them, including details on the sensor, housing, and sharing of relevant software through appropriate repositories is needed. Given the rapid pace of development in accelerometers, we find that manuals for commercially available devices are often unavailable or incomplete. We encourage collaboration between researchers and manufacturers to ensure relevant details and manuals are available in a version-controlled and persistent location (e.g., Zenodo or Dryad).

The values of triaxial acceleration recorded by an accelerometer result from a combination of whole body and local movements [37]. This means that, for the same motion or behaviour, the resulting triaxial acceleration recorded by the device will differ depending on where the sensor is mounted [30]. Changing **attachment location** can shift signal amplitude and axis-specific contributions, which in turn alters derived metrics and behavioural-classification [30, 37]. As a result, reporting the precise anatomical location and the sensor orientation relative to the animal is essential for interpreting results or replicating placements. For example, Harvey-Carroll et al. [30] reported significantly improved accuracy in behavioural classification when accelerometers were positioned on the third scute relative to the first scute of sea turtles. Additionally, Garde et al. [37] reported higher DBA values when accelerometers were placed on the upper back of pigeons (*Columba livia*) relative to the lower back.

The way an accelerometer is attached affects mechanical coupling between the animal and the sensor [6]. Popular **attachment methods** include adhesive (e.g., tape, glue, or epoxy), collars, harnesses, ear tags, and bolts [38]. The degree to which the device can move independently of the animal is critical to reporting as this can introduce relative motion, enhancing or dampening acceleration on one or more axes. Aspects like tightness, intermediate padding, or any modification of the device exterior such as protective casing are also vital in reporting [6, 38, 39]. For example, Martin et al. [40] showed that activity data from accelerometers attached loosely and firmly to dog (*Canis familiaris*) collars was poorly correlated.

It is generally recommended, where possible, to use a ridged attachment to reduce variation in the axes, and also minimize any relative motion between the device and the animal [6, 39]. In cases where fixed devices are not appropriate due to an animal’s anatomy or behaviour, high accuracy in behavioural classification can still be achieved through relatively loose attachments [22, 41]. Additional considerations may arise based on method of locomotion (e.g., walking, swimming, or flying). Attachment choices can change the animal’s movement through altering aerodynamic or hydrodynamic profile or by causing discomfort [30, 34, 38]. This will indirectly change the signal that is being measured.

As a result of differences in device model, attachment location, or attachment methods, accelerometer outputs are unlikely to be directly comparable between studies unless an effort is made to replicate all these elements, which is only possible with access to detailed methodology.

### Deployment settings

**Sampling frequency** determines which behaviours are observable within data [27, 42]. Low sampling frequency risks missing extreme values of acceleration. High sampling frequencies are more likely to capture rapid behaviours but can have negative consequences for data storage space and battery life [27, 42]. Yu et al. (2023) assessed the impact of sampling frequency on behavioural classification in pied flycatchers (*Ficedula hypoleuca*). Rapid transient behaviours required a higher sampling frequency (100 Hz) than longer-duration behaviours (12.5 Hz). Acceleration signals associated with swallowing became aliased when data were subsampled to 25 Hz and 12.5 Hz, preventing reliable detection of this behaviour [27].

Previously, it has been recommended sampling frequency follow the Nyquist–Shannon sampling theorem. That is, the accelerometer should sample at a frequency at least twice that of the fastest movement of interest to reduce signal aliasing and associated distortion [6, 18, 27]. However, appropriate sampling frequency depends greatly on the research question. Yu et al. [27] found that using at least twice the Nyquist frequency was necessary to capture sufficient signal representation (frequency and amplitude) of behaviours. For short-burst behavioural classification and energetic estimations 1.4 times the Nyquist frequency was required. If sampling frequency is known, down-sampling of high frequency data can be applied to harmonise with datasets collected at lower frequencies [43–45].

The **dynamic range** of an accelerometer defines the range of acceleration values that the sensor can measure before saturation [46]. If the dynamic range is set too low relative to an animal’s maximum acceleration, high-acceleration events will not be recorded, leading to systematic underestimation. Using an inappropriately high dynamic range reduces measurement resolution and sensitivity to subtle movements [18]. Accelerometer based studies can include an initial step of determining maximum acceleration prior to deployment so that an appropriate dynamic range can be selected [30].

In addition to dynamic range, some devices allow for a user defined sensor/bit **resolution**. This alters the quality of the logged data, with lower bit resolution potentially precluding key information [18]. Although we are not aware of any empirical studies which have investigated this parameter, its effect can be illustrated mathematically. For example, if a device with a dynamic range of ± 3 g has an 8-bit resolution, a total of 2^8^ “levels” of measurement between − 3 and + 3 g are possible. With a 10 bit resolution, a total of 2^10^ “levels” of measurement are possible between − 3 and + 3 [47]. Higher bit resolution may therefore be particularly important when studying fine-scale behaviours that occur within a narrow acceleration range.

As accelerometers produce a large amount of data, even relative to other forms of biologging device, **onboard processing** can be applied (e.g. to enable satellite transmission over limited bandwidth). Consequently, some devices apply lossy data compression or derive summary metrics (e.g., activity counts or prey capture attempts) before transmitting summary statistics [47]. In our opinion, interpretation of pre-processed data can be challenging, as details are often presented in incomplete or inaccessible formats. It is therefore important that researchers fully understand the output of their equipment [20]. This can only be achieved if manufacturers share detailed information on onboard processing.

### Post processing

**Fig. 2:**
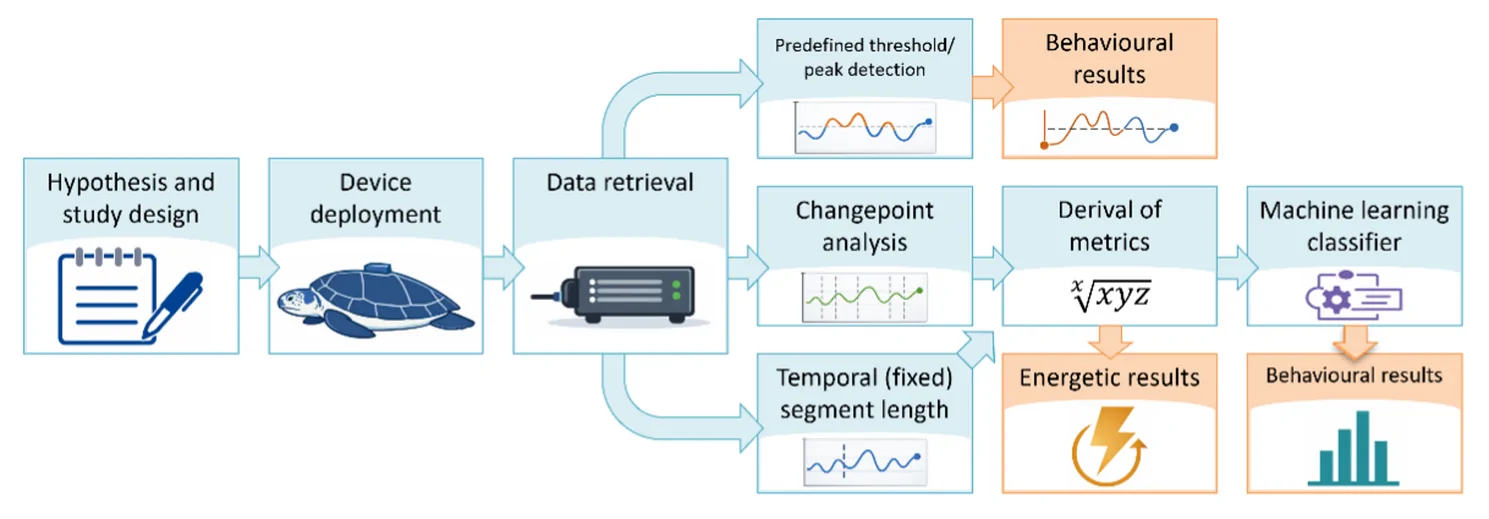
Generalised workflow for accelerometer studies from deployment to data interpretation

Raw accelerometer data typically undergoes segmentation and multiple other post-processing steps prior to analysis [18, 48, 49]. This can substantially influence derived metrics and behavioural inference (Fig. 2). Choices made at this stage can introduce systematic differences between studies and should therefore be reported in sufficient detail to enable replication and comparison.

Data can be segmented based on thresholding or peak detection [23–25]. Alternatively, summary metrics are determined by segmenting acceleration data over discrete time intervals referred to as smoothing windows. **Smoothing window length** can influence subsequent analysis, for example by masking local extreme values [21]. Variable smoothing window lengths are usually identified via change-point analysis [50, 51]. Fixed-time smoothing window lengths should be decided on based on the research question [43, 52]. Larger smoothing windows can capture longer more complex behaviours but can also mask rapid changes in posture or short, high-intensity behaviours [53, 54]. For example, Shepard et al. [21] demonstrated that smoothing window length influenced resulting estimates of ODBA and that the appropriate window length varied among behaviours and species. In Imperial shags (*Phalacrocorax atriceps*), DBA values during flight and running stabilised with running means of ≥ 2 s, whereas swimming stabilised at 2–3 s. Species with slower movement cycles, including whale sharks (*Rhincodon typus*) and leatherback turtles (*Dermochelys coriacea*), required longer smoothing windows of approximately 4 s before DBA estimates stabilised [21].

**Summary metrics** derived from temporally segmented acceleration data vary by study but frequently include minimum, maximum, mean, and standard deviation of acceleration values for all axes, alongside pitch (angle of the surge axes) and roll (angle of the sway axis) within the smoothing window [55]. Composite metrics of DBA are frequently calculated. Prior to calculation of DBA metrics, it is first necessary to isolate dynamic motion from static acceleration, for example using low pass filtering [6, 21, 55]. Overall Dynamic Body Acceleration (ODBA) is calculated as the sum of dynamic acceleration across all axes, while Vectorial Dynamic Body Acceleration (VeDBA) is calculated as the vectorial magnitude of dynamic acceleration. Both ODBA and VeDBA have been used as proxies for energy expenditure, oxygen consumption, and overall activity levels [26].

Increasingly, **behavioural classification** of accelerometer data is performed using machine learning algorithms [19, 56]. Typically, these models are trained using summary metrics (e.g., minimum, maximum, mean, and standard deviation of acceleration along each axis and DBA). If ground truthing data has been collected, this can be used within supervised or semi-supervised models. Unsupervised models can also be used without training data [19, 56, 57]. As machine learning approaches are central to accelerometer-based behavioural inference, there is a growing need for standardised reporting of study design. This is particularly important where models are intended to be reused or transferred between study systems. Without consistent and transparent reporting, model reuse is limited [19].

In a systematic review of studies using supervised machine learning, Wilson et al. [19] reported that 79% lacked sufficient validation procedures to detect overfitting. Their findings reinforce the need for specific reporting standards in accelerometer-based studies to ensure reproducibility and robust ecological inference. The authors outline appropriate validation and reporting of machine learning models in the context of animal-borne accelerometers which we recommend are followed.

The extent to which the above factors influence how well a study can be interpreted or replicated depends heavily on the intended application of the data. Studies aiming to estimate broad activity budgets or relative energy expenditure may be less sensitive to minor differences in attachment or sampling configuration than studies seeking to detect fine-scale or elaborate behaviours [26, 58]. In contrast, studies aiming to quantify specific behaviours may require a higher level of methodological standardisation [19, 26]. It seems likely that apparently minor technical differences could propagate into systematic bias when additional biological, environmental, or analytical variables are introduced [19, 28, 56].

### Case study: sea turtles

To demonstrate the inconsistencies of reporting in accelerometer studies, we present sea turtles as a case study. Accelerometers are increasingly used to study sea turtle behaviour and ecology [43, 59]. The hard carapace of most sea turtles offers an ideal location for attaching biologging devices [60]. In a recently published case report, we aimed to optimise the use of accelerometers attached to loggerhead and green sea turtles to maximise the accuracy of behavioural interpolation while minimising the impact of the device on the animal’s hydrodynamic profile [30]. To do this, we initially compiled details on study design from previous work.

We conducted a review in June 2026 of studies involving the attachment of accelerometers to sea turtles using the Web of Science Database (Clarivate, 2022), searching within all fields, and Google Scholar (Google, 2025), searching anywhere in the text. We searched for the terms (“sea turtle*” OR “Chelonioidea” OR “Cheloniidae”) AND (“accelerometery” OR “accelerometer*”). The review returned 33 results from Web of Science and 557 from Google scholar. Only peer-reviewed studies that involved the attachment of one or more accelerometers directly to a sea turtle were included. Publications that did not involve attaching accelerometers to sea turtles, were not peer-reviewed, or did not make use of the accelerometer were excluded. As result, 40 publications were deemed relevant, with the majority being excluded due to focusing on an incorrect taxon or not using accelerometers. In six cases, studies employed bi-axial accelerometers [23, 61, 62], however these are likely to have the same reporting requirements as triaxial accelerometers and were included in overall summaries. Papers that reanalysed previously collected data were not included.

We determined if each study reported on study species, individual biometrics (mass, curved carapace length, or straight carapace length, all of which are an indication of life-history stage in sea turtles [63, 64]), individual captivity status, attachment location on the animal (with sufficient detail to reproduce deployment), attachment method, device model (or sufficient details to reproduce the device), sampling frequency, dynamic range, parameters sufficient to recreate the segmentation method (e.g., smoothing window length, details on changepoint boundary selection, thresholding, or peak detection), and data resolution (Supplementary Table 1). For each of these methodological parameters, each study was assigned a binary score (1 = reported, 0 = not reported). When variables could be inferred from images (e.g., attachment location from photographs) but were not explicitly reported in the text or standardised diagrams, they were recorded as not reported, as consistency across deployments could not be verified. As it is only likely to be reported when used, onboard processing was not included.

**Fig. 3:**
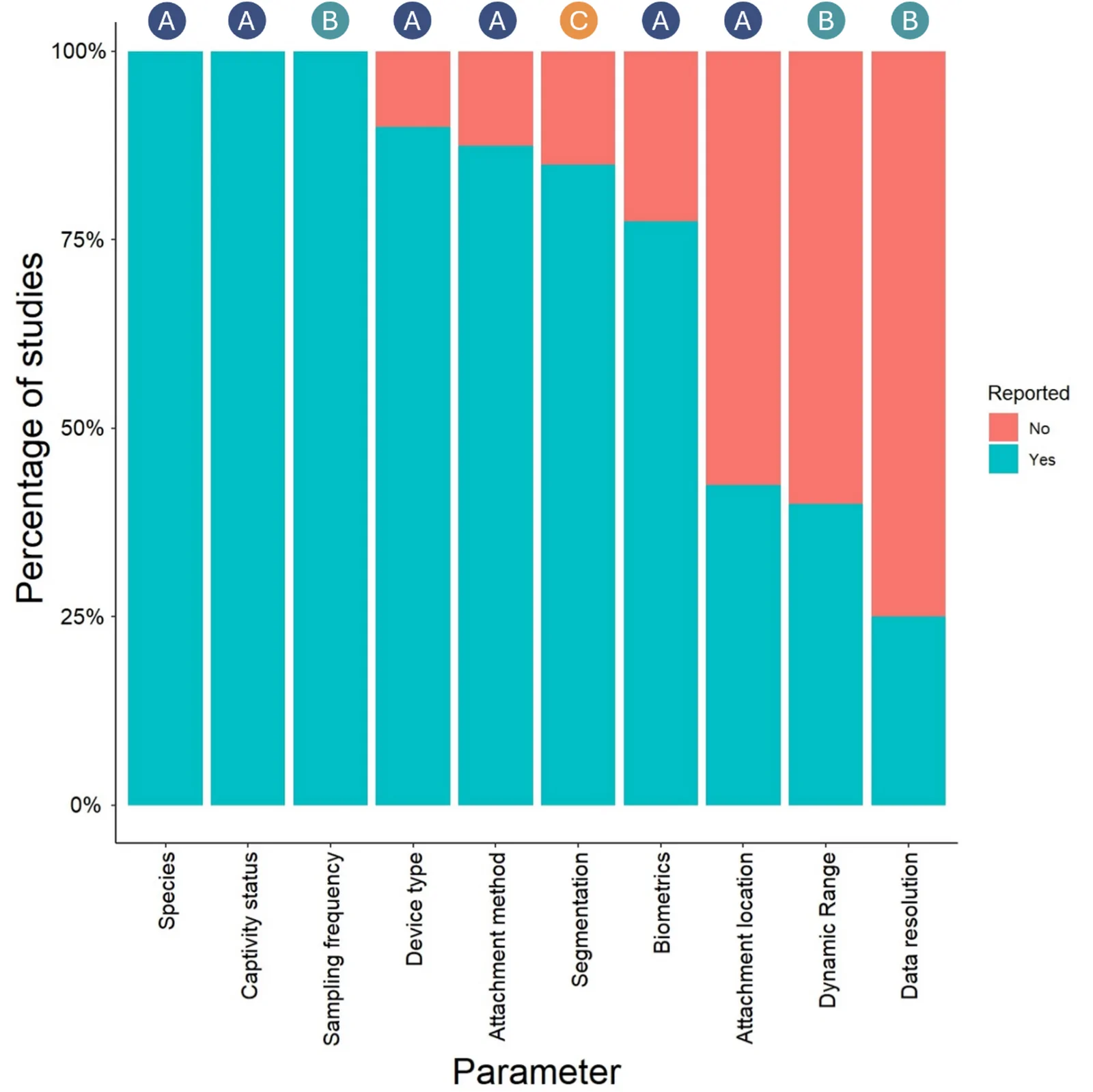
The proportion of studies which involved the attachment of accelerometers to sea turtles which reported (blue) or did not report (red) methodological parameters (Supplementary Table 1). Circles above bars indicate whether the parameters could be considered a physical consideration (**A**), deployment setting (**B**), or a part of post processing (**C**)

Study species, captivity status, and attachment method were reported in all studies. Device description, sampling frequency, detail on segmentation, and individual biometrics were reported in > 75% of studies. Reporting was less consistent for attachment location, dynamic range, and data resolution (reported in < 50% of studies, Fig. 3). Thus, while general study characteristics were routinely documented, key information required for interpreting and reproducing accelerometer measurements was often omitted.

## Discussion and conclusion

Our review of sea turtle studies emphasises important inconsistencies in the reporting of accelerometer-specific methodology. While some methodological parameters were reported consistently (e.g., sampling frequency), others critical for the interpretation, reproducibility, and comparison of studies were frequently omitted. Although our review focused on a single taxa, similar patterns of underreporting are present for other marine and terrestrial taxa [6, 22, 28], suggests that uneven reporting is a widespread issue. With a growing number of publications likely laying foundational work for the field of accelerometery, it is timely that underreporting is addressed.

Critical parameters specifically required for the interpretation of accelerometer studies (e.g., attachment location and dynamic range) were frequently underreported. This presents several key challenges. First, it severely limits reproducibility. Without clear information on attachment location and dynamic range, it is difficult or impossible to replicate study conditions or validate findings using similar methods [2, 3, 18]. Second, the absence of standardised reporting will hamper robust meta-analyses [7, 17, 65]. Comparisons across studies become unreliable when physical differences between devices or differences in device settings are unknown or unaccounted for, potentially confounding results and obscuring ecological patterns. This could be partially overcome through selective meta-analysis design, for example by only comparing effect sizes rather than individual metrics. Finally, as discussed in Payne et al. [10], insufficient reporting poses concerns for animal welfare assessment, particularly in cases when individual mass, attachment location, or attachment method are not reported [7, 66]. Without documentation of attachment methods, and placement, it is difficult to evaluate welfare impacts or to establish best practices for minimising harm.

Although we have identified substantial gaps in methodological reporting, it is not clear from our work, or any previous work we are aware of, the impact that this underreporting may have had on study conclusions. We do not intend to criticise previous foundational work with accelerometers but rather highlight the opportunity to standardise reporting as the field matures.

To counteract these issues, we recommend the adoption of minimum reporting standards in animal-borne accelerometer studies (Table 1). These extend the minimum standards recommended by Bodey et al. [7] (e.g., study species, number of devices deployed, number of tagged animals, mean animal mass, method of attachment, device mass, and length of deployment) to include considerations specific to accelerometers [7]. In addition, we encourage sharing of justifications for parameter selection, as this will benefit the adaptation of protocols to other systems.

**Table 1:** Recommended minimum reporting standards for animal-borne accelerometer studies, expanding on general parameters from [7]

Parameter	Recommended reporting	Example of reproducible reporting
Attachment location	This description should follow available standards for the taxa, with reference to identifiable structures (e.g., specifying a scute in hard-shelled sea turtles), and standard anatomical descriptors where possible (e.g., dorsal, ventral, anterior, posterior), using Nomina Anatomica Veterinaria when appropriate [67], or by providing a clear diagram with an exact standard location marked.	“Centre of the second vertebral scute of a turtle in anterior-posterior direction.” [68] For example diagrams illustrating attachment locations, see Kinoshita et al. or Okuyama et al. [23, 69].
Attachment method	Although well reported generally in biologging studies attachment method may have specific considerations for accelerometer studies [6, 7, 9, 12]. With regards to the interpretation of accelerometer data, the materials used to attach the device and the degree of movement of the device relative to the animal are important to report.	“The tags were mounted on an aluminum release plate (1-mm thick), which in turn was glued to the fur on the seal’s upper back with two-component epoxy (Ergo 7211). For the DTAG-3, a 2.5 mm magnesium nut, a stainless steel washer, and a 5 mm nylon pin (glued to the float) held the tag to the aluminum plate.” [70]
Device model or description	This should include relevant details such as model, mass and, in the case of flying or swimming animals, volume and shape.	“The 2015 tag (DTAG-3) contained a syntactic foam float and had an integrated Argos transmitter and VHF beacon. A more compact version of the tag (DTAG-4) was used in 2016, and this was attached to a high-pressure closed cell foam float containing an Argos transmitter with an integrated low-power UHF beacon (SPOT 6, Wildlife Computers, Seattle, USA). The complete DTAG-3 package measured 55 × 37 × 205 mm and weighed 325 g in air, while the DTAG-4 measured 40 × 33 × 180 mm and weighed 206 g. Both tags were approx. 20 g buoyant in water.” [70]
Sampling frequency	Can be reported in Hz.	“Devices were set to record all three acceleration channels at a frequency of 5Hz.” [71]
Dynamic range	Can be reported on the G full-scale or other appropriate unit.	“Measuring within the range of ± 16 g” [72]
Segmentation method where appropriate	This is highly contextual depending on the approach used, however should contain sufficient detail to repeat the method.	See the *Materials and Methods* sections of Jeantet et al. (2018) for an example of reproducible segmentation of accelerometer data [73].
Data resolution	Can be reported in bits.	“Tri-axial acceleration data were recorded with 22-bit resolution.” [74]

In conclusion, we suggest that the inconsistent reporting of accelerometer settings and placement in animal studies undermines the reproducibility, comparability, and ethical evaluation of biologging research. Accelerometers have specific reporting needs beyond general biologging studies. By adopting a standardised framework for reporting these critical methodological details, the scientific community can enhance transparency, improve the reliability of findings, and support better welfare outcomes for study animals. While this comment focuses on accelerometers, the principles outlined here are broadly applicable across other areas of biologging, including the use of GPS, heart rate monitors, and temperature loggers [7, 10]. Establishing clear reporting standards for specific types of biologgers is a necessary step toward more rigorous, ethical, and impactful research in animal ecology and behaviour.

## Supplementary Information

Below is the link to the electronic supplementary material.

Supplementary Material 1

## Data Availability

All data generated during the literature review are included in Supplementary Table 1.
